# Sarcopenia: Molecular Pathways and Potential Targets for Intervention

**DOI:** 10.3390/ijms21228844

**Published:** 2020-11-22

**Authors:** Jorge Pascual-Fernández, Alejandro Fernández-Montero, Alfredo Córdova-Martínez, Diego Pastor, Alejandro Martínez-Rodríguez, Enrique Roche

**Affiliations:** 1 Medical Extrahospital Emergency Service of Navarra, 31500 Pamplona, Spain; jpfoliva@gmail.com; 2 Prevention and Health Service, University Clinic of Navarra, 31008 Pamplona, Spain; afmontero@unav.es; 3 Biochemistry, Molecular Biology and Physiology, Faculty of Health Sciences, GIR Physical Exercise and Aging, University of Valladolid, Campus Duques de Soria, 42004 Soria, Spain; a.cordova@uva.es; 4 Department of Sport Sciences, University Miguel Hernández (Elche), 03202 Alicante, Spain; dpastor@umh.es; 5 Department of Analytical Chemistry, Nutrition and Food Sciences, Faculty of Sciences, University of Alicante, 3690 Alicante, Spain; amartinezrodriguez@gcloud.ua.es; 6 Alicante Institute for Health and Biomedical Research (ISABIAL), 03010 Alicante, Spain; 7 Department of Applied Biology-Nutrition, Institute of Bioengineering, University Miguel Hernández, 03202 Elche, Spain; 8 CIBER Fisiopatología de la Obesidad y Nutrición (CIBEROBN), Instituto de Salud Carlos III (ISCIII), 28029 Madrid, Spain

**Keywords:** aging, inflammation, oxidative stress, physical activity, sarcopenia, satellite cells

## Abstract

Aging is associated with sarcopenia. The loss of strength results in decreased muscle mass and motor function. This process accelerates the progressive muscle deterioration observed in older adults, favoring the presence of debilitating pathologies. In addition, sarcopenia leads to a decrease in quality of life, significantly affecting self-sufficiency. Altogether, these results in an increase in economic resources from the National Health Systems devoted to mitigating this problem in the elderly, particularly in developed countries. Different etiological determinants are involved in the progression of the disease, including: neurological factors, endocrine alterations, as well as nutritional and lifestyle changes related to the adoption of more sedentary habits. Molecular and cellular mechanisms have not been clearly characterized, resulting in the absence of an effective treatment for sarcopenia. Nevertheless, physical activity seems to be the sole strategy to delay sarcopenia and its symptoms. The present review intends to bring together the data explaining how physical activity modulates at a molecular and cellular level all factors that predispose or favor the progression of this deteriorating pathology.

## 1. Introduction

Aging is associated with a loss of muscle strength (sarcopenia), leading to muscle mass loss, and resulting in decreased motor function [1]. This inevitable process leads older people to a progressive deterioration that can give rise to debilitating pathologies. Muscle mass loss leads to a decrease in quality of life, affecting their overall well-being and self-sufficiency [2]. Furthermore, sarcopenia is one of the multiple scenarios that contribute to the physiological and cognitive deterioration that appear during senescence progression. Furthermore, this degenerative process can exacerbate other underlying conditions, and ultimately cause a decrease in life expectancy [2,3].

General deterioration starts in the fifth decade of life and muscle loss is estimated at approximately 0.8% per year [4]. Other authors have estimated a gradual decline in skeletal muscle mass and strength of around 2% per year starting from the sixth decade of life [5]. The loss of muscle mass generally occurs due to the combination of two factors: muscle atrophy and the death of muscle cells, resulting both in loss of strength and muscle mass [6,7]. At a molecular level, an altered expression of protein synthesis and degradation factors have been observed [8]. In addition, sarcopenia reduces the cross-section of muscle fibers, due to their deterioration and loss of motor units [6,7]. Molecular mechanisms are not yet well defined, being an intense area of research [9,10,11,12,13].

The cause for sarcopenia onset is multifactorial, including neurological factors related to the loss of motor neurons, endocrine alterations resulting from the decreased or loss of hormone expression (such as testosterone or growth hormone (GH)), loss of muscle motor units, and finally, nutritional and lifestyle changes related to the adoption of sedentary habits [14,15,16]. Therefore, sarcopenia appears at a moment in life when physical activity decreases considerably [16], which could be one of the main triggers for the aforementioned events. There is an estimated 30% reduction in muscle mass in 80-year-old adults compared to 20-year-olds, and a 20% reduction in muscle area. Changes are observed both in fiber size and number, particularly type II fibers [17].

The lack of physical activity favors loss of muscle strength and mass [18,19]. Therefore, it can be considered that exercise protects against sarcopenia onset. However, there is little consensus as to what are the most recommended types of exercise in order to prevent or treat sarcopenia. On one side, aerobic physical activities such as walking and running improve maximum O_2_ consumption (VO_2_max). On the other hand, strength exercises help to improve muscle function and neuromuscular adaptation. Both exercise types are known to help decrease morbidity and mortality [20,21].

Inactivity is generally accompanied by an unbalanced diet rich in saturated fat, leading to increased fat deposits in the adipose tissue, liver, and muscle. Therefore, a high-fat diet can affect the composition and structure of skeletal muscle and satellite cells (SC). These cells are responsible for post-exercise muscle repair and regeneration processes. In non-obese individuals, skeletal muscle mass can account for 40% of total body weight, and contributes to optimal metabolic control, as well as regulate glucose and fatty acid circulating levels by modulating their absorption, usage, and storage [22]. In this context, excess fatty deposits affect the skeletal muscle by altering the hepatic growth factor (HGF) signaling pathway. This protein is attached to the extracellular matrix and is released after performing physical activity to repair the damage caused to the tissue during exercise, activating the SC. Therefore, the lack of physical activity in conjunction with an unbalanced diet are key factors that promote obesity and fat infiltration in muscle fibers and liver, altering the response of SC to HGF [23]. In addition, nitric oxide (NO) production increases with physical activity, and is a key signal in HGF activation. Therefore, the alteration of HGF signaling could result in a lack of SC activation and thus incorrect repair of muscle fibers and myogenesis [24,25,26,27,28,29]. Lack of physical exercise or disuse would lead to a decrease in NO production and subsequently affect the release of HGF from the extracellular matrix. This will in turn affect SC, which will remain in the G0 phase of the cell cycle. This process could worsen with aging, resulting in sarcopenic obesity associated with SC apoptosis [25].

Altogether, sarcopenia, or muscle mass loss, is a consequence of atrophy from disuse. However, the molecular mechanisms underlying this phenomenon are unknown. Alterations in the transcriptional regulation at the myocyte level have been proposed, triggering the activation of proteolytic processes [30]. One of the main pathways involved in the degradation of muscle proteins is the ubiquitin-proteasome system. This system plays a key role in controlling muscle fiber size. In this degradation mechanism, specific ligases bind ubiquitin to substrate proteins to prime them for proteolysis. Preliminary studies indicate that two ubiquitin ligases are upregulated when muscle atrophy appears; atrogin-1 and muscle-1 ring-finger protein-1 (MuRF-1). The expression of both proteins also increases in autophagy-related muscle degeneration processes. In addition, MuRF-1 may also be associated with mechanisms that cause neuromuscular junction alterations [31,32,33]. In this context, the Foxo family of transcriptional factors, which integrates signals generated by nutrient deprivation and stress situations, appears to be involved in the expression of genes related to the degradation systems of ubiquitin-proteasome and autophagy pathways, activating atrogin-1 and MuRF-1 and triggering the degradation of muscle proteins [34].

In addition, the role of myostatin should be considered. Myostatin (also known as GDF8) is an extracellular messenger of the transforming growth factor superfamily (TGF-β), which inhibits muscle growth. Myostatin controls the cell cycle, inhibiting the factors that regulate myogenesis [35]. The mechanism by which myostatin exerts its effects is similar to the rest of the TGF family members; that is, it forms tetrameric complexes consisting of two type I and 2 type II receptors [36]. In most cases, the ligand binds to type II receptors, subsequently recruiting type I receptors. The formation of this complex allows to phosphorylate serine and threonine residues in type I receptors through the kinase activity of type II receptors. In the case of myostatin, its activation requires the participation of ActRIIB (activin receptor IIB) and ALK4 (activin-like kinase 4), both of which are also involved in activin signaling [36]. The aged muscle expresses increased myostatin signaling and consequently initiates a process of progressive atrophy [36,37,38]. On the other hand, when myostatin signaling is blocked, this results in increased protein synthesis and muscle strength.

Therefore, there is a lack of consensus among the published studies, most likely due to the different experimental protocols applied. Nevertheless, it seems clear that the lack of SC activity (due to incorrect HGF signaling), together with an increase in intramuscular proteolytic activity (involving atrogin-1 and MuRF-1 activation) and an inhibited myogenesis (caused by increased myostatin signaling), play important roles in the decrease in strength and muscle mass during the aging process.

Altogether, sarcopenia seems to be a multifactorial process (Table 1), which will be further detailed in this review. As there is currently no cure for sarcopenia, the most efficient strategy is to take preventive measures to delay its progression. There is increasing evidence that maintaining physical activity throughout life delays its onset. Therefore, evidence regarding the role of physical activity in delaying sarcopenia and its symptoms will also be presented.

## 2. Satellite Cells

SC are located between the basal lamina and sarcolemma within the muscle fibers, being in a state of mitotic quiescence and, at the metabolic level, in a state of semi-latency [24]. These cells are activated and proliferate in response to stimuli such as physical exercise, injuries, or mechanical stress. In this context, SC are the main contributors to muscle maintenance, repair, and growth [25,26,27]. In order to carry out muscle regeneration growth or repair, SC must follow a three-step process [28,29,39,40]:

1. Activation from their quiescence state.
2. Proliferation by entry into the cell cycle.
3. Differentiation and fusion to form multinucleated myotubes, by activating protein synthesis.

SC activation involves a signaling route in which calmodulin-Ca binds to nitric oxide synthase (NOS), which results in NO production. This pathway is activated in the muscle in response to exercise, injury, denervation or trauma associated with intense muscle activity [26,41]. As previously mentioned, NO favors the release of HGF bound to the extracellular matrix by mechanisms yet to be elucidated. In turn, HGF promotes SC activation [42,43,44]. On the other hand, the inhibition of NO production could lead to a lower release of HGF, and therefore less activation of SC [43]. The SC activation results in muscle fiber regeneration and repair, through SC fusion with myoblasts or activating their proliferation [45].

Throughout the aging process, many of the factors that promote the activation of SC are greatly diminished or even absent. Thus, the SC activation pathway initiated by NO appears to be less efficient in the elderly. The study conducted by Leiter et al. [46] demonstrated that SC of young mice are activated immediately after a stimulus, while SC of older mice may have a 24-h activation delay. Similarly, mechanical stimuli response is also affected. For example, a longer stretching of muscle fiber must be performed in order to activate the SC [26,46]. Otherwise said, more intense stimuli are required for SC activation in elder animals. Interestingly, in atrophy by disuse, muscle fibers acquire characteristics very similar to aged fibers, leading both situations to significant levels of muscle disability [45,46]. Therefore, aging is associated with a significant decrease in the number of active SC.

## 3. Oxidative Stress

As previously mentioned, sarcopenia involves the participation of various processes that need to be analyzed in detail in order to have a global view of the problem. One of these refers to the presence of oxidative stress. Numerous scientific studies have established, in a well-documented way, the role played by reactive oxygen (ROS) and nitrogen species (RNS), collectively known as RONS, in numerous biological and pathological processes. ROS include superoxide anion (O_2_^.−^), hydroxyl radical (HO^.^), along with non-radical species such as hydrogen peroxide (H_2_O_2_). Among RNS are mainly NO and peroxinitrite anion (ONOO^−^), both free radicals. The most important production of ROS in muscle cells is located in mitochondria. Nevertheless, NO is mainly produced by NOS isoenzymes, in which arginine as a substrate is transformed into citrulline [47,48,49,50].

The most widely accepted theory about aging is that this process involves an uncontrolled participation of free radicals. ROS, which appear as collateral products of mitochondrial metabolism, would cause progressive damage to key cell macromolecules (lipids, proteins, and DNA), altering both their structure and function. These oxidized molecules would accumulate and spread oxidative damage due to a malfunction of antioxidant and repair systems. Due to its high metabolic rate, the skeletal muscle is especially susceptible. For this reason, muscle cells of older individuals are predisposed to accumulate oxidized molecules and therefore exposed often to oxidative stress, compared to young individuals. A plausible hypothesis to explain the lack of response to oxidative stress observed in aged muscle tissue could be its inability to recognize redox signaling molecules, necessary to activate genes that encode antioxidant and repair proteins [49].

In this context, the skeletal muscle is the most abundant tissue in the human body, and where the majority of the oxidation metabolic reactions occur. In addition, the muscles easily adapt in response to different stimuli, including an excess of RONS production [51]. Since this response is mitigated in aged muscle tissue, we can hypothesize that the overproduction of RONS together with a decreased response from antioxidant systems could contribute to the appearance of sarcopenia and delay of muscle regeneration in the elderly [52].

Excessive generation of RONS causes alterations related to energy metabolism and muscle contraction function, both in the coupling excitation-contraction, in the ion homeostasis, as well as in the contraction/relaxation process of muscle fibers [53,54]. Thus, in the muscle, the highest demand for energy is produced when muscle contractions are activated. The production of energy in the mitochondria produces radical O_2_^−^, as a collateral product of the electron transport chain, being able to produce other RONS [50]. However, RONS produced during muscle contraction can trigger an adaptive response, inducing the activation of transcription factors that modulate the gene expression of antioxidant enzymes, such as superoxide dismutase, involved in O_2_^−^ elimination. Therefore, RONS can act as mediators in various cell signaling pathways that would culminate in cellular responses to both physiological and pathological situations. Therefore, cells need to maintain the balance between the generation of RONS and its elimination. An imbalance towards increased production of RONS at the intracellular level could trigger a situation of oxidative stress. Therefore, cellular redox regulation in the skeletal muscle is critical for its homeostasis [55].

In addition, NADPH-oxidase, located in the plasma membrane and at the level of the transverse tubules of the skeletal muscle, is a key element in redox signaling. NADPH-oxidase is formed by several subunits, distributed between the cytoplasm and the plasma membrane in inactive cells. The enzyme catalyzes NADPH oxidation to NADP^+^, using molecular oxygen as substrate and leading to O_2_^−^ production. Several studies show that skeletal muscle cells release O_2_^−^ to extracellular space during contraction [55,56,57]. This radical should play an instrumental role in redox signaling, modulating cytoprotective responses that help to maintain cellular viability [58,59]. Therefore, redox signaling errors should be considered as key molecular factors in sarcopenia development.

## 4. Neuronal Factors

Muscle mass loss partly explains the loss of strength related to age, giving relevance to the concept of muscle quality [60]. However, several neurological factors are also partly responsible for the loss of strength [61].

Muscle contraction is a complex process coordinated by various interrelated brain structures. In order to carry out this motor control, the motor area must be integrated into complex circuits with the prefrontal cortex, basal ganglia, and cerebellum. Unfortunately, this set of structures suffers to a greater extent the consequences of the aging process, becoming atrophic with time [62].

At the spinal cord level, two processes limit the structure necessary for muscle contraction. On one hand, starting at the age of 60, around a quarter to half of the motor neurons are lost [63]. Second, there is a reduction in axonal size and its myelinization at the level of the peripheral nerve [64], with the consequent loss of nerve conduction velocities [65].

Motor neuron loss reduces the estimated number of motor units determined by electromyography [65]. This loss of motor neurons leads to the denervation of muscle fibers. As a result, neural plasticity is activated, remodeling the innervation of motor units while increasing their size during the process. This helps to maintain muscle function to some extent. Therefore, the loss of strength occurs later and does not coincide with the decreased motor units [65]. For example, Urbanchek et al. [66] observed in rats that only 11% of the strength decrease observed was due to the denervated fibers, suggesting that additional factors contribute to the development of sarcopenia. On the other hand, the capacity and duration of the opening of the sodium channels located in the motor plate is affected with age. This process results in strength loss and fatigue [67].

In addition, the loss of motor neurons appears to occur more often in the larger motor neurons, which are the largest motor units that connect to fast fibers [61]. This focus implies a loss of fast muscle fibers, increasing reinnervation by small motor neurons that turn fast muscle fibers into slow ones. This process explains the different percentages in the types of muscle fibers observed with age [68].

Finally, the expression of neurotrophic factors related to the nervous system, such as brain-derived neurotrophic factor (BDNF), a neurotrophin associated with synaptic plasticity and cell survival, are reduced with age [69]. BDNF has been described to be involved in the metabolic processes of peripheral systems such as muscle fibers. BDNF release is stimulated by skeletal muscle contraction, improving fat oxidation metabolism and muscle fiber functionality during the contraction process [70]. In addition, BDNF could stimulate the DNA repair process by activating the cyclic-AMP response element binding protein (CREB), although the effects of BDNF signaling on skeletal muscle are not yet clear [70]. Nevertheless, it has been observed that, in the absence of BDNF, the muscle loses its ability to synthesize proteins, and reduces myogenic regulatory factors, linked to muscle regeneration and development, leading to muscular atrophy [71]. In this context, the production and release of brain BDNF increases with physical activity [72,73,74]. This process could improve the homeostasis of the central nervous system and the skeletal muscle, delaying the onset of sarcopenia [70,75].

## 5. Inflammatory Processes

The immune system plays an important role in skeletal muscle regeneration. Sarcopenia leads to a reduction in muscle regeneration capacity caused by local inflammatory mechanisms (especially due to eccentric exercises). This induces an alteration of the neuro-hormonal response, which results in an imbalance in protein synthesis and degradation. This is mainly mediated by interleukin-1β (IL-1β), tumor necrosis factor-α (TNF-α), interleukin-15 (IL-15), and insulin-like growth factor-1 (IGF-1). The activation of the inflammatory process results in a decrease in the number of SC [67]. Therefore, an important part of the changes that occur during aging, including immuno-senescence, can be explained as a consequence of the imbalance between pro-inflammatory and anti-inflammatory processes, resulting in a chronic low-grade muscle pro-inflammatory state, which in the long term can progressively lead to muscle deterioration [76].

TNF-α activates local vascular endothelial cells, which causes NO release that in turn increases vascular permeability, allowing the passage of pro-inflammatory cells and triggering inflammation. Therefore, TNF-α and its soluble receptors are important markers of muscle loss and strength in the aged muscle during the latent inflammatory process [76]. Thus, an increased plasma concentration of TNF-α correlates with lower muscle mass and strength, contributing to sarcopenia onset. Increased concentration of TNF-α is also related to the activation of apoptosis in muscle cells [77].

Another key cytokine present in senescence processes is interleukin-6 (IL-6). High plasma levels of this cytokine correlate with fatigue and disability, associated with muscle destruction [78]. Therefore, it is believed that IL-6 circulating levels are intimately associated with decreased age-related muscle function, and loss of mass and strength. In this sense, the release of IL-6 and TNF-α and its increase in serum would indicate a general pro-inflammatory state that could be active throughout the aging process, favoring the onset of sarcopenia [79].

Considering cytokines as targets for therapeutic strategies in sarcopenia, certain treatments have been developed that act upon the TNF-α signaling pathways. The results have not been very promising, hence processes activated by these cytokines have been investigated as alternative targets, for example the inducible form of NOS (iNOS) [80]. In this context, the effects of cytokine-induced oxidative stress depend on increased iNOS expression. Inhibitors of iNOS activity have demonstrated a clear efficiency to prevent symptoms of muscle wasting [81].

As previously mentioned, the aging process is related to increases in pro-inflammatory cytokines such as IL-6 and TNF-α, resulting in an increased production of acute-phase reactive proteins by the liver, such as C-reactive protein (CRP) or α1-antichimotrypsin (ACT). High levels of IL-6 and/or CRP are associated with a decrease in physical performance and a greater degree of disability [82]. On the other hand, the acute muscular regenerative response is modulated by macrophages. In this sense, it has been observed that a greater degree of neutrophilia, which affect the appropriate temporal macrophage phenotype transition, is associated with a decrease or even the inhibition of muscle regenerative capacity [83].

Finally, it has also been observed that the evolution of sarcopenia is accelerated in the presence of chronic inflammatory pathological states such as those observed in insulin resistance, atherosclerosis, and Alzheimer disease. Older adults are more prone to develop these pathologies compared with their younger counterparts under similar environmental conditions. Chronic inflammation states observed in these pathologies present an increase in circulating pro-inflammatory cytokines, increased presence of dysfunctional T-regulatory cells and a T-cell-senescent phenotype. These alterations compromise the immune response, resulting in a condition known as “immune-senescence” or “inflammaging”. In this context, increased levels of ACT have been shown to be associated with Alzheimer’s disease [82]. Another example is provided by older patients with HIV in which chronic inflammation favors the development of other pathologies, including cardiovascular, kidney or liver disease, cancers, certain neurological diseases and “geriatric syndromes”, including sarcopenia, frailty, and falls [84].

## 6. New Perspectives to Focus Sarcopenia Management

**Figure 1:**
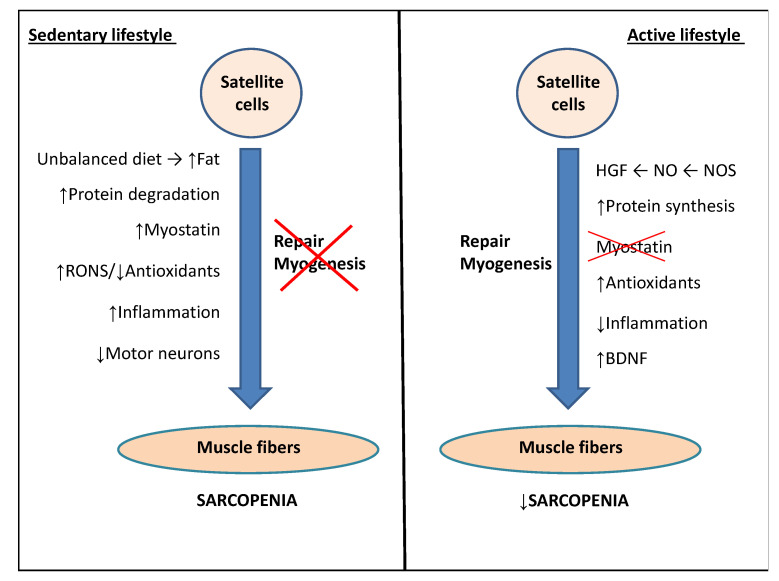
Scheme of the main factors predisposing to sarcopenia and the role of physical activity in delaying this process. See text for more details. Abbreviations and symbols used: BDNF, brain-derived neurotrophic factor; HGF, hepatic growth factor; NO, nitric oxide; NOS, nitric oxide synthase; RONS, reactive oxygen and nitrogen species; (↑) increased; (↓) decreased.

Since a cure for sarcopenia does not exist, all studies on delaying its onset and lessening its symptoms have one thing in common: physical activity. Sedentary lifestyles in older adults cause skeletal muscle inactivity, which accelerates strength loss and therefore muscle atrophy. The recovery of muscle mass would be difficult, since efficient protocols to reach an optimal previous situation have not been implemented [85,86]. Muscle disuse would also reduce the rate of postprandial amino acid muscle absorption, resulting in a decrease in myofibrillary protein synthesis [87,88]. In this negative scenario, exercise would compensate for the harmful effects caused by aging such as sarcopenia, modulating all the factors involved: oxidative stress, neural dysfunction, and inflammation (Figure 1). In addition, exercise is beneficial to prevent obesity and mitochondrial dysfunction, which enhance sarcopenia progression.

### 6.1. Role of Physical Activity in Modulating Oxidative Stress to Delay the Sarcopenia Associated with Aging

Physical activity has been shown to cause changes in antioxidant enzyme levels, both in acute bouts and over time. As discussed above, these modifications can be triggered by ROS generated during exercise, which are key elements for the onset of muscle adaptation to delay sarcopenia. Thus, post-exercise ROS in muscle cells can act as triggers or repressors for a wide variety of genes [55]. In this context, the skeletal muscle has the ability to adapt to certain stimuli or stressors caused during concentric or eccentric muscle traction, stretching or contraction.

A key issue would be to identify the most efficient exercise type in delaying sarcopenia. Resistance exercises seem to be associated with a lower use of aerobic metabolism and oxidative processes when compared to demanding aerobic exercises [50]. On the other hand, aerobic training increases NO levels, as a result of increased NOS. This, in turn, favors blood flow in muscle fibers due to the vasodilation produced. This process indirectly protects the muscle from pro-oxidant effects that appear during exercise, allowing for a greater arrival of circulating antioxidants [89].

As previously mentioned, many studies have proposed that ROS production during exercise constitutes a natural stimulus that leads to an improvement of antioxidant defenses and decreased activation of the inflammatory pathways, resulting in a greater resistance of organisms to free radicals [90,91]. This strongly indicates that, despite new discoveries and the development of new drugs, non-pharmacological therapies such as physical exercise and nutritional support could be considered the basis for the prevention and treatment of muscle abnormalities associated with age.

Adaptive changes in antioxidant protective processes have been detected in both endurance and resistance exercises, such as increased activity of catalase (CAT) and superoxide dismutase (SOD). The increase in CAT and SOD are induced by an increased production of H_2_O_2_ and O_2_^−^, respectively, during exercise [91]. On the other hand, glutathione peroxidase (GPX) is an enzyme with five isoforms that catalyzes the reduction of H_2_O_2_ into H_2_O, as well as organic hydroperoxides (ROOH) and hydroxylated derivatives (ROH). This antioxidant enzyme uses reduced glutathione (GSH) that is transformed into oxidized glutathione (GSSG). GSSG cannot be released to the cytosol and must be reduced back to GSH in the mitochondrial matrix. Meanwhile, GSH levels in the skeletal muscle appear to be constant [92] while GSSG levels increase with age. Subsequently, the GSH/GSSG ratio decreases significantly, suggesting that aging could cause significant alterations in glutathione balance in skeletal muscle [93].

Altogether, oxidative stress parameters show that exercise is able to promote a protective and adaptive effect against increased ROS, reducing the risks of cell damage. This can be achieved with aerobic as well as resistance training routines [74,91,93] and this could be a beneficial strategy to delay sarcopenia.

### 6.2. Role of Physical Exercise in Modulating Satellite Cell Activity to Delay Sarcopenia Associated with Aging

Exercise increases the expression of the neuronal form of NOS (nNOS) in the muscle, leading to the activation of SC. Furthermore, exercise decreases the expression of myostatin in muscle fibers, as observed in individuals that followed an exercise plan, while this was not observed in a sedentary control group. All of this leads to the thought that SC can be activated mechanically, depending on age and muscle type [3]. With regard to the expression of myostatin, it has been observed that exercise decreases the expression of this muscle growth-inhibiting messenger, regardless of gender or age, although certain exceptions have been observed in older women [94].

In a study with 8-month-old mice, SC activation was found to be triggered by NO produced by nNOS in the quadriceps muscle. Activation occurred exclusively in this muscle when it was exercised, while passive quadriceps did not display SC activation. However, muscle type should be considered in this context, since muscles of the lower body do not respond in the same way as those in the upper body segment [42,95]. In addition, no increased nNOS activity was observed in aging muscles, explaining the poor activation of SC with age. Therefore, all this evidence might suggest that the activation of SC and nNOS expression would depend specifically on the type of muscle, the workload applied and the individual’s age.

Research evidence indicates that resistance training promotes SC activation and proliferation. The SC response to exercise occurs in both young and old individuals, although the level of response differs among the age groups [96]. Also, it has been observed that certain individuals have a higher SC response than other, leading to the thought that there are responders and non-responders, possibly depending on genetic factors [97,98].

Certain research has quantified the increase in SC in the skeletal muscle after a period of resistance training, with a 19% increase after 30 working days and 31% after 90 days of training. This proliferation of SC coincides with an increase in mRNA of two cell cycle markers: cyclin-D1 and p21 [99]. In addition, changes in SC following adaptations caused by exercise are associated with a decrease in O_2_ levels in muscle cells and increased levels of vascular endothelial growth factor (VEGF) and epidermal growth factor (EGF), all of which can influence SC proliferation induced by resistance training [100,101].

Another key factor that will affect SC proliferation is the decrease in telomere length throughout life. This leads to the impossibility of SC division, once telomeres have reached a critical length. Interestingly, training routines accompanied by moderate oxidative stress can positively influence a compatible length of telomeres, both in leukocytes and skeletal muscle [102,103]. The presented evidence strongly indicates that a regulated activity of SC is going to be critical for sarcopenia delay, due to the role they play in the adaptation to exercise, strength maintenance, and functionality of the skeletal muscle.

### 6.3. Role of Nutrition to Delay Sarcopenia Associated with Aging

Recent publications have shown how certain nutritional interventions, such as high protein or essential amino acid and leucine ingestion, in combination with resistance training, may help to delay the fiber loss observed in sarcopenia [104,105,106,107,108]. Muscle protein could be considered as a key mechanical component and an amino acid reserve for several body functions. The amount and quality of protein ingested in diet is a very important factor for muscle mass anabolism. Meanwhile, dietary recommendations for protein consumption in adults are established at around 0.8 g/kg/day, while the European Society for Clinical Nutrition and Metabolism (ESPEN) recommends over 1 g/kg/day (up to 1.5 g/kg/day) for older adults in order to delay the increased risk of sarcopenia [104,105,106,107,109]. 

However, the elderly usually present a low nutrient intake (including proteins) due to several reasons, such as physical disability and lack of appetite, being exacerbated by a poor access to quality foods due to economic limitations [3,105,106]. In addition, certain individuals reduce or eliminate protein intake, especially from meat, due to chewing and swallowing difficulties. This leads them on many occasions to choose other foods with less protein content or poor amino acid quality. For this reason, it is necessary to present alternative high-quality protein sources, such as eggs or fish that are easy to chew; as well as modifications in culinary preparations, in order to increase the proportion of soft textures [104,105,106]. In addition, to achieve the daily recommended protein intake, older adults need to eat protein at every meal to promote muscle anabolism. Avoiding these recommendations can give rise to chronic protein malnutrition, which has been shown to be associated with an approximately four-fold increased risk of developing severe sarcopenia [3,104].

### 6.4. Role of Biological Rhythms to Delay Sarcopenia Associated with Aging

Recent evidence suggests that the circadian clock is instrumental in promoting skeletal muscle growth and body homeostasis. Therefore, the maintenance of biological rhythms could be essential to prevent or delay sarcopenia. Although the circadian rhythms are generally regulated by the cycles of light/darkness, there are other secondary agents, such as physical activity, that can contribute to its modulation. In this sense, the molecular clock is essential in the circadian coordination of specific genes expressed in the skeletal muscle, such as those controlling structure, function, and metabolism. Maintaining a circadian muscular rhythm favors muscle growth, while clock disruption favors sarcopenia [110].

## 7. Conclusions

From a public health perspective, physical exercise can be considered as a non-pharmacological strategy to improve the quality of life of the elderly and delay sarcopenia onset [111]. Currently, there are several lines of research to determine the biochemical and physiological mechanisms involved in age-induced muscle deterioration. The implications in the myolysis process are so varied that it is difficult to define what are the determining factors and reactions that converge in these processes. However, it seems right to say that in almost all of these mechanisms, exercise acts as a key facilitator of muscle regeneration and repair processes, delaying sarcopenia.

Exercise benefits skeletal muscle homeostasis through changes in muscle fiber composition that results in improved muscle performance. As commented throughout the present review, exercise helps modulate sarcopenia progression by controlling oxidative stress and SC activity. Under these principles, an effective physical intervention would improve muscle strength and quality. In addition, physical activity protocols should be performed in conjunction with an adequate diet to avoid inflammation, and taking in consideration the circadian rhythm. Under these principles and following the principles of training, the programs should include 2–3 days per week of strength training to improve the response and muscular adaptations. Aerobic programs to maintain cardiovascular function can also be added alternatively throughout the rest of the week. One day for resting and mental recovery is highly recommended. Resistance as well as endurance programs must follow the recommendations of a professional that will prepare the exercise protocols adapted to each particular individual taking in consideration motor limitations, intensity and time involved.

## Figures and Tables

**Table 1:** Etiopathological factors around sarcopenia.

**Malnutrition:**
Increased intramuscular saturated fat deposition.
Increased risk of atherosclerosis.
**Alterations in muscle structure:**
Lower activity of satellite cells caused by defects in HGF signaling.
Decrease in motor units caused by intramuscular proteolysis activation.
Presence of oxidative stress.
**Altered muscle signaling:**
Activation of myostatin (GDF8) pathway.
Decreased presence of anabolic hormones: Testosterone, growth hormone.
Neuronal dysfunction.
Increase in pro-inflammatory cytokines: TNF-α, IL-1β, and IL-6.
**Decreased physical activity.**

Abbreviations used: IL, interleukin; TNF, tumor necrosis factor.

## Abbreviations

ACT	α1-antichymotrypsin
ACtRIIB	Activin receptor IIB
ALK4	Activin-like kinase 4
BDNF	Brain-derived neurotrophic factor
CAT	Catalase
CREB	Cyclic-AMP response element binding protein
CRP	C-reactive protein
EGF	Epidermal growth factor
GH	Growth hormone
GPX	Glutathione peroxidase
GSH	Reduced glutathione
GSSG	Oxidized glutathione
H_2_O_2_	Hydrogen peroxide
HGF	Hepatic growth factor
HIV	Human immunodeficiency virus
HO.	Hydroxyl radical
IGF-1	Insulin-like growth factor-1
IL	Interleukin
iNOS	Inducible form of nitric oxide synthase
LD	Linear dichroism
MuRF-1	Muscle-1 ring-finger protein-1
nNOS	neuronal form of nitric oxide synthase
NO	Nitric oxide
NOS	Nitric oxide synthase
O_2_^.−^	Superoxide anion
ONOO^−^	Peroxinitrite anion
RNS	Reactive nitrogen species
ROH	Organic hydroxylated derivatives
ROS	Reactive oxygen species
RONS	Reactive oxygen and nitrogen species
ROOH	Organic hydroperoxides
SC	Satellite cells
SOD	Superoxide dismutase
VEGF	Vascular endothelial growth factor
TGF-β	Transforming growth factor-β
TNF-α	Tumor necrosis factor-α
